# Biorefining of platinum group metals from model waste solutions into catalytically active bimetallic nanoparticles

**DOI:** 10.1111/1751-7915.13030

**Published:** 2017-12-28

**Authors:** Angela J. Murray, Ju Zhu, Joe Wood, Lynne E. Macaskie

**Affiliations:** ^1^ School of Biosciences University of Birmingham Edgbaston Birmingham B15 2TT UK; ^2^ School of Chemical Engineering University of Birmingham Edgbaston Birmingham B15 2TT UK

## Abstract

Bacteria can fabricate platinum group metal (PGM) catalysts cheaply, a key consideration of industrial processes and waste decontaminations. Biorecovery of PGMs from wastes is promising but PGM leachates made from metallic scraps are acidic. A two‐step biosynthesis ‘pre‐seeds’ metallic deposits onto bacterial cells benignly; chemical reduction of subsequent metal from acidic solution via the seeds makes bioscaffolded nanoparticles (NPs). Cells of *Escherichia coli* were seeded using Pd(II) or Pt(IV) and exposed to a mixed Pd(II)/Pt(IV) model solution under H_2_ to make bimetallic catalyst. Its catalytic activity was assessed in the reduction of Cr(VI), with 2 wt% or 5 wt% preloading of Pd giving the best catalytic activity, while 1 wt% seeds gave a poorer catalyst. Use of Pt seeds gave less effective catalyst in the final bimetallic catalyst, attributed to fewer and larger initial seeds as shown by electron microscopy, which also showed a different pattern of Pd and Pt deposition. Bimetallic catalyst (using cells preloaded with 2 wt% Pd) was used in the hydrogenation of soybean oil which was enhanced by ~fourfold using the bimetallic catalyst made from a model waste solution as compared to 2 wt% Pd preloaded cells alone, with a similar selectivity to *cis* C18:1 product as found using a Pd‐Al_2_O_3_ commercial catalyst.

## Introduction

Platinum group metals (PGMs) are widely used in many industries due to their distinctive properties such as catalytic activity, chemical inertness, corrosion resistance and thermoelectric stability (see Dong *et al*., 2015). In general, the global demand for primary PGMs is increasing due to their wide applications. A major use is in automobile catalytic converters to reduce toxic exhaust fumes (Osterauer *et al*., 2011; Wiseman and Zereini, 2011; Almeida *et al*., 2016). The catalysts are routinely ‘thrifted’ by varying the catalytic composition according to the PGM market price (Mouza *et al*., 1995; Xiao and Laplante, 2004) but PGM loadings on catalytic converters are unlikely to decrease further overall due to stringent vehicle emissions regulations (Bloxham, 2009; Yang, 2009).

It was reported by Johnson Matthey that in 2012, the gross demand for Pt, Pd and Rh in car catalytic converters was 97.2 tons (over 44% of gross Pt demand), 216.8 tons (72% of gross Pd demand) and 24.9 tons (78% of gross Rh demand), respectively, and the recycled Pt, Pd and Rh from these catalysts were 39.7 tons, 57.9 tons and 8.7 tons respectively (Dong *et al*., 2015). Net PGM demands are still likely to be high in the future due to increasing vehicle numbers and environmental pressures.

However, vehicle exhaust gases contain small particles of PGMs due to abrasion from the support of these autocatalysts, becoming deposited within road dust (Schafer and Puchelt, 1998; Cinti *et al*., 2002) to, in some instances, concentrations similar to those found in low grade ores (Jackson *et al*., 2007). Road dust can therefore potentially be considered as an ‘urban mine’ where the considerable energy demand of comminution incurred in traditional mining from a primary ore is forestalled due to the powdery nature of the material (Murray, 2012). Traditional mining operations also use a significant amount of electricity for the transport of personnel, material and ore, powering production machines and mineral processing, as well as for vital health and safety‐related applications such as the pumping of water, ventilation and refrigeration (Krogscheepers and Gossel, 2015).

Various methods exist to ‘upgrade’ the PGM content of road dust to bring the PGM content to a level that becomes economic to extract. However, at scale, upgrading of these powdery materials to economic PGM levels still presents challenges (Murray, 2010, 2012). Various leaching methods to extract PGMs from solid wastes have been described, for example, microwave‐assisted leaching reduces both the volume of acid and the time needed for PGM recovery from wastes (see Patel and Dawson, 2015 for review), but the scalability of microwave‐assisted technologies for bulk materials like crushed primary ores and road dust, and the scope for continuous operation, still remains to be established.

For the next generation of ‘clean’ hydrogen‐powered vehicles, fuel cells are used to convert hydrogen fuel into power. The anodic and cathodic reactions of low‐temperature hydrogen fuel cells use PGM electrocatalysts. With finite resources, this will introduce conflicting demands between today's transport requirements and tomorrow's need for carbon neutral transport.

Road dust from vehicles, once washed into gullies, becomes dispersed into the environment and waterways (Prichard, 2008) with PGMs either attached to larger fragments of the catalytic converter or released as individual detached (< 0.3 μm) nanoparticles (Prichard, 2012). These can be regarded as non‐recoverable once dispersion has occurred. On the other hand, the fate of metallic nanoparticles (NPs) in the environment is a concern (e.g. review by Ek *et al*., 2004; Hristozov and Malsch, 2009), with potential impacts on agriculture, for example as shown using engineered metallic NPs from solids from a wastewater treatment plant (Judy *et al*., 2015). Hence, to mitigate against potential environmental hazards and to safeguard future supplies of PGMs for fuel cells, as well as to reduce the environmental burden of PGM primary processing from ores, it is becoming mandatory to recover and reuse precious metals effectively and sustainably (Anon, 2008).

With automotive catalysts as an example (being the source material for road dust prior to environmental PGM dispersion), a new approach was pioneered to recover PGMs from acidic spent automotive catalyst leachates using cells of the bacterium *Desulfovibrio desulfuricans* (Yong *et al*., 2003). This organism deposits precious metals from solution via their bioreduction into metallic nanoparticles (NPs) held on the bacterial surface. This method was originally developed simply for PGM recovery from waste leachates but later work showed that biorecovered PGMs from an industrial waste could be used as a catalyst in a low‐temperature fuel cell (Yong *et al*., 2010), that is, a ‘one‐pot conversion’ of wastes into new catalyst material, which forms the focus of this work.

Many other bacteria, including *Escherichia coli,* reduce PGMs enzymatically onto their surface through hydrogenase activity (see Deplanche *et al*., 2014) amongst other methods for making bio‐supported metallic nanoparticles (Singh, 2015). The resulting metallic NPs are held tightly on the bacterial cells; hence, a supported, stabilized ‘bionanocatalyst’ can be produced, having a wide range of potential catalytic applications (see Deplanche *et al*., 2011a, 2014 and references therein). These include commercially relevant synthetic chemistry, (Deplanche *et al*., 2014), sometimes with better product selectivity in comparison with established catalysts (Zhu, 2014). Bacterially supported catalyst is recoverable in repeated reaction cycles without attrition or loss (Bennett *et al*., 2013), and hence, this approach is beneficial in terms of catalyst conservation and reduced wastage, especially where the catalyst is self‐immobilized onto a tightly adhering biofilm for application in a continuous process (Yong *et al*., 2015).

Biorecovery of platinum group metals from wastes to produce neo‐catalysts (Mabbett *et al*., 2006; Murray *et al*., 2007; Macaskie *et al*., 2011) is particularly attractive for low‐value or sacrificial applications, for example clean‐up of groundwater with respect to chlorinated aromatic pollutants (Deplanche *et al*., 2008), decontamination of pesticides (Mertens *et al*., 2007), treatment of oils to remove toxic contaminants (Macaskie *et al*., 2012) or in catalytic oil upgrading (Omajali *et al*., 2015, 2017) as well as in high‐value ‘green chemistry’ applications (e.g. Deplanche *et al*., 2014).

This approach pioneers a new area of environmental nanotechnology where the potential hazards of nanoparticle migration (Lead and Valsami‐Jones, 2014; Valsami‐Jones and Lynch, 2015) are forestalled by the retention of the catalytic NPs onto the micron‐sized, robust ‘carrier’ bacterial cells and macroscopic biofilm, where the catalyst ensemble remains tightly bound (Yong *et al*., 2015).

Many PGM wastes are aggressive (e.g. those resulting from acid dissolution), and here the potential for enzymatic bio‐nanoparticle synthesis is more limited. To overcome this, a two‐step approach was developed whereby initially the bacteria reduced small amounts of (e.g.) Pd(II) to Pd(0) ‘seeds’ enzymatically; the ‘seeds’ then functioned as chemical catalysts in the subsequent reduction and recovery of PGMs from acidic solutions such as leachates from industrial waste (Mabbett *et al*., 2006), electronic scrap (Creamer *et al*., 2006), refractory brick furnace linings (Murray *et al*., 2007) and spent automotive catalysts (Murray *et al*., 2017) as well as road dust (Murray *et al*., 2015). Notably, the metallic composition of the recovered catalytic material reflected the composition of the metals in the mixture (Macaskie *et al*., 2011) as shown by X‐ray diffraction analysis of bulk samples (Murray *et al*., 2007) as well as by direct analysis of the solid using highly sensitive proton‐induced X‐ray emission analysis of specimen microareas (Mabbett *et al*., 2006).

A mass loading of 5 wt% of Pd(0) onto cells is often adopted for catalytic conversions (e.g. Creamer *et al*., 2007; Deplanche *et al*., 2014). Reduction of the Pd(0) loading on *Desulfovibrio* cells from 5% to 2% mass of Pd(0) gave ~30% less activity in the reduction of ${\text{CrO}}_{4}^{2-}$ (Skibar *et al*., 2005) as well as in the hydrogenation of itaconic acid (Creamer *et al*., 2007). Hence, the need to conserve PGM resources and reduce cost conflicts with the requirement for optimal catalytic activity. We propose that using the ‘seeding’ method, additional metal could be deposited from waste to bring the metal loading up to the optimal level for a particular reaction. Proof of this concept formed the objective of this work, which compared the activity of bacteria additionally metallized from a model solution to those bearing only the initial ‘seeds’. Previous work has focused on Pd (e.g. Creamer *et al*., 2007; Deplanche *et al*., 2014; Zhu *et al*., 2016) although many PGM wastes contain both Pd and Pt (Ek *et al*., 2004). Hence, cells in this work were ‘seeded’ using both Pd and Pt to various loadings prior to metal removal from model metal mixtures of Pd and Pt. The use of this approach to make catalysts successfully from a real waste leachate was shown previously (using cells ‘seeded’ with 5 wt% Pd) where the extent of ${\text{CrO}}_{4}^{2-}$ reduction was comparable using model and real waste leachates‐derived catalysts (Murray *et al*., 2017). Initially, we focused on reduction of Cr(VI) for comparison with previous work. Other studies have established the utility of this bimetallic catalyst for making sacrificial catalyst for upgrading of heavy oil (Omajali *et al*., 2017). To assess the future potential for this approach in wider applications in oil processing into higher value materials, bimetallic catalysts were evaluated with respect to their ability to hydrogenate soybean oil. Here, not only the conversion rate but the product selectivity is important. Catalytic selectivity towards the desired *cis*‐fatty acid products is an outstanding problem in the hydrogenation process of vegetable oils despite long‐standing research on palladium catalysts. The *cis*‐product is key in industrial hydrogenations such as olefin metathesis (Molnar *et al*., 2001). The ability of a biogenic bimetallic catalyst to achieve this goal would realize a dual benefit, and hence the selectivity of the soybean oil hydrogenation with the Pd/Pt bio‐catalyst was also evaluated.

## Results and Discussion

### Reduction of Pd(II) and Pt(IV) using cells preloaded with Pd(0) and Pt(0)

The yellow target mixed metal solution (0.34 mM Pt(IV) and 0.42 mM Pd(II)) before reduction had an absorbance maximum of 401 nm and a pure Pt solution a maximum of 463 nm. Hence, A_419_ was used to assess initially the reduction of metals from solution. The colour of the solution was an accurate representation of metal concentration as established by representative samples analysed using the SnCl_2_ method which was, in turn, previously cross‐validated polarographically (Mikheenko, 2004).

**Figure 1:**
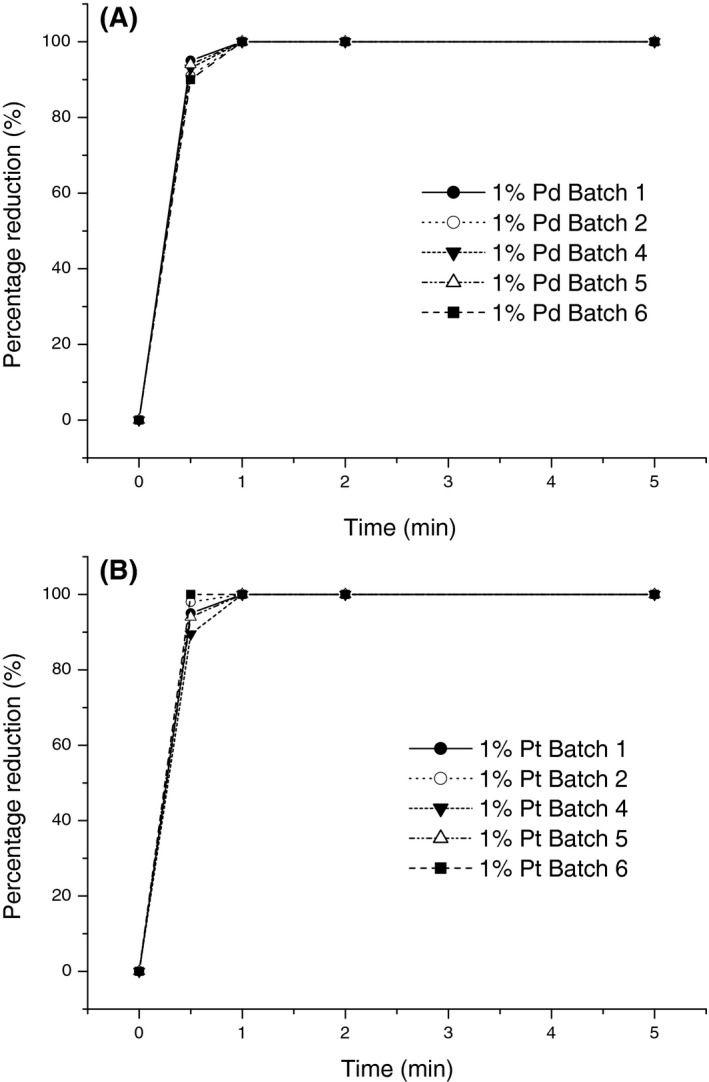
Reduction of target metals from solution using cells preloaded at (A) 1 wt% Pd; (B) 1 wt% Pt. Results obtained using cells preloaded at 2% and 5% metal were identical to those shown. Results are shown for five independent preparations (●,○,▼, ▵, ■).

The reduction of target metals by cells preloaded with Pd or with Pt at all three preloadings was examined. The interbatch variability was insignificant, and the target metal reduction profiles using Pd and Pt preloaded cells at all three preloadings were very similar, with example data shown in Fig. 1. The target metals were removed from solution within 2 min under H_2_ (Fig. 1). All of the metals were reduced onto the cells (by assay of the spent solution), and previous studies have established that the composition of PGM material deposited on the cells via the seeds reflects the original composition of the metal mixture (Mabbett *et al*., 2006; Murray *et al*., 2007). The seeding step is important for the patterning of the initial Pd‐nuclei (and hence to obtain good dispersion of the subsequent metal deposits). The seeding is performed physiologically, via the activities of hydrogenases (Mikheenko *et al*., 2008; Deplanche *et al*., 2010). Allowing cells to deposit metals directly from a waste without this prepatterning resulted in a few, large metal deposits at the cell surface (Yong *et al*., 2015) probably attributable to inactivation of hydrogenases (and other metal reductases) at the low pH of the waste solution. The Pd of the latter cells had reduced catalytic activity (Yong *et al*., 2015).

From the results of this study, an economic consideration would indicate use of 1% metal preloaded cells to be preferable as this shows an almost identical PGM reduction rate to that of the higher loadings (Fig. 1) with minimal ‘sacrificial’ metal required in the ‘seeding’ step. Pd would be the ‘seeding’ metal of choice as it is generally significantly cheaper than Pt. However, this economic consideration does not take into account the effect of seeding on the subsequent catalytic activity of the resultant bimetallic catalyst which is assessed below.

### Catalytic activity of the recovered material in the reduction of Cr(VI)

Material from five batches (identical as shown in Fig. 1) was combined according to their metal loading (samples A–F: Table 1) to assess the best bimetallic catalyst with respect to reduction of Cr(VI) as percentage reduction of 0.5 mM ${\text{CrO}}_{4}^{2-}$ against time.

**Table 1:** Catalyst preparations used in this study

Preparation	Initial loading^a^	Final loading (wt%)	Total metal (wt%)^a^
A	1% Pd	6.6% Pd/8.4% Pt	15%
B	2% Pd	7.6% Pd/8.4% Pt	16%
C	5% Pd	11% Pd/9% Pt	20%
D	1% Pt	5.6% Pd/9.4% Pt	15%
E	2% Pt	5.6% Pd/10.4% Pt	16%
F	5% Pt	6.0% Pd/14% Pt	20%

^a^Proportions are in wt% not atomic ratios; atom amount of Pd is approx. twice as much as that of Pt (respective atomic weights are 106.4 and 195.09).

**Figure 2:**
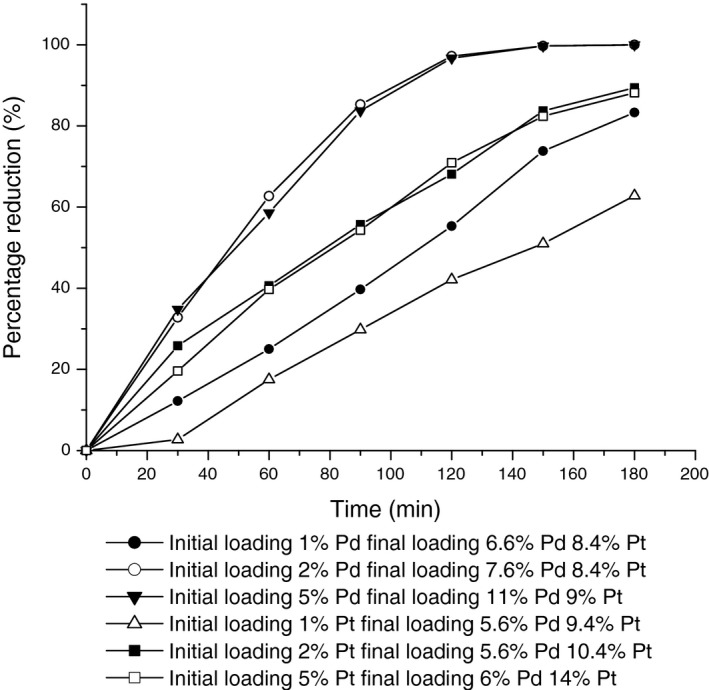
Catalytic activity of the metal loaded cells in the reduction of Cr(VI). Cells were preloaded with either Pd or Pt to 1 wt%, 2 wt% or 5 wt% as shown and then further loaded from the mixed metal challenge solution to make the final catalyst as shown in Table 1. Data are means ± SEM from five independent preparations of each. □: Commercial 2 wt% Pd/Al_2_O_3_. Samples were: A (●); B (○); C (▼); D (▵); E (■); F (□).

It was observed (Fig. 2) that where Pt was the predominant metal in the bimetallic catalyst Cr(VI) was not reduced fully, even after 3 h (samples D,E,F). Preparations seeded with 2 wt% or 5 wt% Pd fully reduced Cr(VI) in under 3 h (samples B and C). A 1% Pd preloaded sample that was predominantly Pt (sample A) showed 20% residual unreacted Cr(VI) after 3 h. Catalysts with the highest initial Pd content (samples B and C) were more active; note that the 1 wt% preloaded Pd samples had a rate of Cr(VI) reduction of ~half that of those preloaded at 2 wt% and 5 wt% (Fig. 2). Hence, despite the higher potential economic benefit of using the lowest amount of Pd in the seeding step, this would be compromised by the lower activity of the final catalyst. The 2 wt% and 5 wt% Pd preloaded catalysts gave overall reduction rates of 2.77 μmoles mg metal^−1^ min^−1^.

In contrast to preloading with Pd, the Cr(VI) reduction test (Fig. 2) suggests that cells initially loaded with Pt are less suitable. Tests using Pt loading only (i.e. without additional Pd) were not performed but the poorest sample preloaded with Pt (1%) was the least active, with Cr(VI) reduction being ~50% complete after 160 h, whereas the 1% Pd preloaded counterpart had achieved > 70% reduction by this time (Fig. 2). There was no apparent ‘rescue’ by additional Pd deposited from the metal mixture following 1% Pt preloading as Pt preloaded samples containing final amounts of 5.6% and 6% Pd (2 wt% and 5 wt% Pt preloading) both catalysed Cr(VI) reduction more effectively than the 1% Pt preloaded samples even though the latter also contained 6% Pd (Fig. 2).

These preliminary studies illustrate the potential for biomanufacturing new bimetallic Pd/Pt catalysts from wastes, although additional metals are likely to be present in real wastes (Macaskie *et al*., 2011) and become incorporated into the catalyst (Mabbett *et al*., 2006; Murray *et al*., 2007). To check these results, parallel work (Murray *et al*., 2017) used the same biomaterials, divided between the two studies (5 wt% Pd seeds; sample C in Table 1) to show that a similar result was obtained to that reported here, using a catalyst prepared from spent automotive catalyst leachate; the rate of Cr(VI) reduction was ~30% slower using the bimetallic catalyst made from waste, possibly due to ‘dilution’ of the Pd/Pt with other components. For example, the real waste leachate contained some Rh (~10%) as well as Fe; the possible effect of ‘diluent’ metals was not evaluated. The structure of the bimetallic catalyst was not examined but, given the promising results described here and confirmed using real waste (Murray *et al*., 2017), this warrants further study. For example, other studies (Deplanche *et al*., 2012) showed that *E. coli* preloaded with Pd(0) and then allowed to reduce Au(III) manufactures core‐shell structures by a route which involves migration of re‐oxidized Pd(II) through/around the nascent Au(0) to become re‐reduced at the surface of the nanoparticle as a Pd(0) ‘shell’. It is worth noting that the Au/Pd core‐shell structures were catalytically more active (in an oxidation reaction) than either metal alone (Deplanche *et al*., 2011b). In other work, mixtures of Pd/Pt were more promising than monometallic bionanocatalysts in catalytic upgrading of heavy oil from the Canadian oilsands (Omajali *et al*., 2017), and the activity was retained using bimetallic catalyst sourced from a simulated road dust leachate as a potential route to economic manufacturing (Murray *et al*., 2015).

Importantly, while ${\text{CrO}}_{4}^{2-}$ is a charged substrate, oils are hydrophobic and hence further studies addressed whether the catalytic activity of the bimetallic would extend to processing of commercially relevant oils of vegetable origin.

### Catalytic activity of the recovered material in the hydrogenation of soybean oil

The above studies suggest the potential for using this approach in the remediation of a toxic ionic contaminant (${\text{CrO}}_{4}^{2-}$). However, the catalytic activity using a fully dissociated (anionic) substrate in aqueous solution is not necessarily predictive of the activity against other substrates. As an example, the bio‐Pd of a mutant strain of *Desulfovibrio fructosovorans* (with altered hydrogenases) showed enhanced activity in reduction of the small, readily water‐soluble ${\text{CrO}}_{4}^{2-}$ ion but less difference was seen between the bio‐Pd of the parent and mutant strain with respect to hydrogenation of the larger, less dissociated itaconic acid molecule (Skibar *et al*., 2005). This may suggest a limitation of transport of larger, less charged molecules within/across the hydrogel of the periplasmic space, that is, the catalytic activity observed would be dependent on the location of the hydrogenases (which mediate Pd(II) reduction) and also the nature of the substrate molecule. As all four hydrogenases in *E. coli* (as used in this study for the ‘seeding’ reactions) are cytoplasmic membrane‐bound (i.e. internal to the periplasmic space, with two of them facing outward into it), it cannot be assumed that the same pattern of catalysis would be followed as with bio‐Pd of *Desulfovibrio* spp. Hence, the *E. coli* bimetallic catalyst was evaluated in a hydrogenation reaction using soybean oil, which comprised ~50% C18:2 (derived as linoleic acid: Zhu *et al*., 2016). The initial oil contained no *trans* C18:1 (derived as elaidic acid) and ~23% *cis* C18:1 (derived as oleic acid). The conversion of C18:2 is shown in Table 2. Whereas a commercial catalyst achieved 95% conversion after 1 h, this was only ~10% using 2 wt% Pd‐seeded cells alone. However, the conversion was increased by ~fourfold using the bimetallic catalyst (Table 2), the reaction being ~91% complete after 2 h and at a rate ~2.5‐fold slower than using a commercial catalyst (Table 2). The selectivity towards the desired product (*cis* C18:1) was the same (30%) at the 50% conversion stage with the commercial and bimetallic catalysts (not shown). Hence, although the bimetallic catalyst gave a slower rate than the commercial catalyst, the reaction was not compromised in terms of selectivity for the desired product. Given that the penetration of the large molecules in soybean oil into the cells is not known, this result may imply conversion at the catalyst surface. In this respect, Redwood *et al*. (2008) suggested that fragments of the bacterial outer membrane may act as an amphipathic ‘bridge’ to a hydrophobic substrate in the non‐aqueous phase. Hence, the ‘presentation’ of the bimetallic catalyst to its substrate may be a key factor in the observed activity.

**Table 2:** Catalytic activity of Pd catalysts in the hydrogenation of soybean oil C18:2

Catalyst	Conversion^a^ @ 1 h	Conversion^a^ @ 2 h
2% wt Pd/Al_2_O_3_	95%	100%
2% wt Pd on *E. coli*	10%	22%
2 wt%Pd then to 16 wt % mixed Pd/Pt on *E. coli* b	38%	91%

Linoleic acid (C18:2) was the main fatty acid derivatized from the soybean oil by assay, comprising ~50% of the total.

a Conversion is loss of the initial C18:2 acid (%) over time. The initial concentrations of (derivatized) *cis*‐C18:1 (oleic acid) and *trans*‐C18:1 (elaidic acid) were ~23% and zero respectively. At the 50% conversion stage, the percentage of oleic acid was similar with the three catalysts (30–35%). Data are means from two independent experiments.

b Sample B in Table 1.

### Examination of the seeded cells by TEM

**Figure 3:**
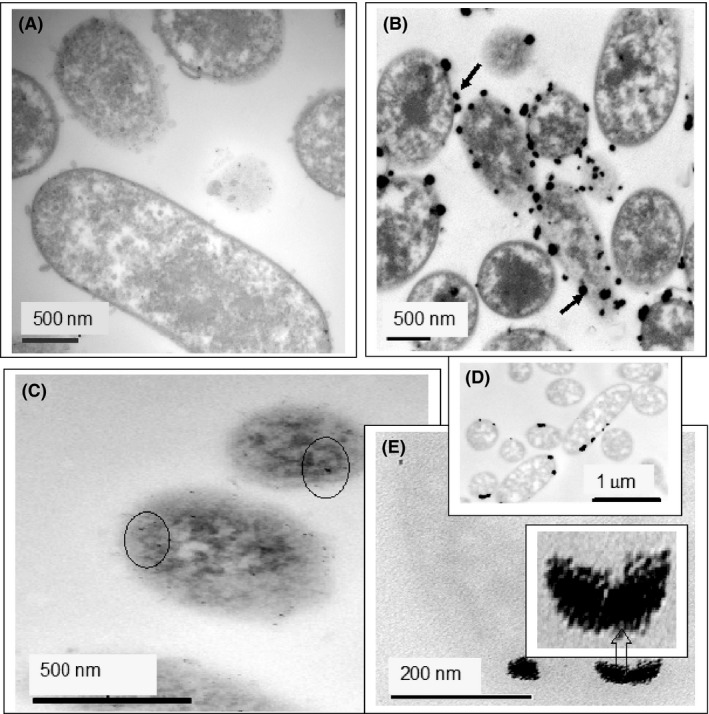
*Escherichia coli* cells before loading with metal (A) and the bimetallic catalyst made from mixed metal solution (B). The increased metal loading is seen as more numerous large deposits extruded from or standing proud of the cells (arrowed) as compared to the Pd‐seeded cells (C). Preloading with Pd to 5% of the bacterial dry weight (C) shows that individual nanoparticles are generally too small to resolve but occasional NPs are visible (circled). (D) Cells preloaded with 5% Pt. (E) Single cell showing intracellular deposits of Pt with NPs comprising clusters of very small NPs, some twinned (inset).

TEM imaging was used to visualize the cell‐bound metals. The example of Fig. 3A shows osmium‐stained cells unchallenged with Pd, while Fig. 3C shows cells preloaded with 5 wt% Pd and Fig. 3B shows the same preparation after reducing the target metal mixture onto the Pd(0)‐ ‘seeds’. Cells before metal exposure showed no electron‐opaque nanoparticles (NPs) (Fig. 3A) whereas the 5 wt% Pd preloaded cells (Fig. 3C) showed very small NPs at the cell surface, plus some NPs located intracellularly. At 5%, mass loading of Pd resolution of individual NPs is difficult; high‐resolution images with elemental mapping show very small Pd‐nanoparticles at the cell surface and throughout the cells (Fig. S1). Similarly, the formation of small intracellular Pd‐NPs by *Desulfovibrio* was shown by Omajali *et al*. (2015) and was apparent also in electron micrographs of *E. coli* (Foulkes *et al*., 2011) where the cells retained sufficient metabolic activity to be used in a tandem biotransformation together with Pd‐mediated catalysis. In the presence of osmium stain (to add contrast), the Pd deposits are difficult to resolve (Fig. 3C) and osmium was omitted from the platinum‐treated samples. The Pt‐seeded cells (Fig. 3D,E), unstained with Os, were pale and indistinct; many appeared to be naked while some had electron‐opaque deposits at the cell surface (Fig. 3D). Some cells showed large intracellular deposits (Fig. 3E). Moreover, the intracellular Pt‐NPs apparently comprised agglomerations of small NPs either as discrete entities and/or showing some morphological features (Fig. 3E).

The mechanism of intracellular Pt deposition in *E. coli* remains to be established. A comprehensive study by Maes *et al*. (2017) showed that the patterning of Pt metallic deposits on/in bacterial cells was species‐dependent and was also influenced by the speciation of the Pt supplied to the cells (Pt(II), Pt(IV), chloride or amine complexes and the pharmaceutical complexes cisplatin and carboplatin); this complexity further argues against a utility for Pt as a seeding metal.

The overall outcome of pre‐seeding with Pt in this study was to reduce the efficacy of the catalysts that had been ‘seeded’ from Pt(IV) (above) as compared to Pd(II). In contrast to Pd, as the cells that had accumulated Pt showed few, heavy deposits (Fig. 3D,E), fewer but larger seeds, would have offered less opportunity for an even distribution of additional metal in the secondary loading step from the metal mixture. The cellular distribution of metals from model and actual waste solutions has not been examined in detail. However, much of the additional metal appeared to be deposited at the cell surface (Fig. 3B). A similar effect was seen in the deposition of Pd/Pt from an industrial processing waste onto cells of the related *Serratia* sp. (Yong *et al*., 2015). These had ~half the activity in the reduction of Cr(VI) as using pure Pd(0) but the extent to which this was attributable to the NP pattering as compared to the effect of the bimetallic nature of the catalyst was not examined.

In the case of Pd‐seeded cells in this study (Fig. 3B), and similarly to that reported by Yong *et al*. (2015), after additional loading with the target metal mixture, the deposits were larger (~50 nm; Fig. 3B). The larger NPs were located mainly at the cell surface and were also visible as cell outgrowths (Fig. 3B). The surface ‘array’ of the finished bimetallic catalyst would be expected to facilitate activity against substrates which are too large to enter the cells and, indeed, which are presented as hydrophobic substrates (see above).

## Conclusions

This study optimized the cell preloading of Pd and Pt ‘seeds’ for the purpose of subsequent metal recovery from solutions of mixed target metals in a model system. Cells of *E. coli* MC4100 with a 2 wt% Pd preloading offered the best option when taking into account initial metal reduction from solution, the subsequent catalytic activity after target metal recovery from solution and cost of production using Pd(II) to form the seeds. This combination achieved full reduction of target metals in 2 min and was the most catalytically active using ${\text{CrO}}_{4}^{2-}$ to assess catalytic activity against a charged substrate. The bimetallic catalyst was also effective in the selective hydrogenation of soybean oil, conferring a ~fourfold increase in conversion over that obtained using 2 wt% Pd(0) on bacterial cells unsupplemented with additional metals. Application in a non‐polar, hydrophobic system has potential for use in chemical synthesis reactions, where membrane residua may play an amphipathic role. A full evaluation and life cycle analysis of ‘neo‐catalyst from waste’ is now required. Towards this, commercial potential has been shown for bio‐Pd in the Heck and Suzuki chemical coupling reactions (Deplanche *et al*., 2014), while Pd/Pt biorecovered from automotive catalyst waste leachate has been applied successfully in the selective hydrogenation of 2‐pentyne (Murray *et al*., 2017).

## Experimental procedures

### Growth of organisms

*Escherichia coli* MC4100 cells were cultured in 12 litres of nutrient broth under anaerobic conditions (Deplanche *et al*., 2008, 2010). Cells were harvested by centrifugation, washed three times in 20 mM MOPS‐NaOH buffer pH 7.0 and resuspended in a known volume of buffer. The cell density was checked by OD_600_ which was converted to bacterial dry weight by a previously determined calibration. With a dry weight of cells between 20 and 30 mg ml^−1^, the cell suspensions were then split into six aliquots in preparation for premetallization.

### Premetallization of cells

Solutions of 2 mM Pd(II) or Pt(IV) were prepared in 1 mM HNO_3_ using Na_2_PdCl_4_ and K_2_PtCl_6_ salts respectively. The required volume of solution was added to aliquots of cells to achieve the desired varied metal loading (1%, 2% or 5% by mass). The shaken mixture was allowed to stand (15–30 min) for biosorption of the metals. Hydrogenase activity was retained during this period (by assay), and cells retained membrane integrity as shown by flow cytometry (I.P. Mikheenko and L.E. Macaskie, unpublished). H_2_ was bubbled through the suspensions (30 min; 30°C) for reduction of metal onto the cells. Metal reduction/removal was confirmed in sample supernatants using a SnCl_2_‐based assay (Creamer *et al*., 2008). Following full reduction of metals (within 30 min), the ‘seeded’ cells were harvested centrifugally, washed and resuspended in distilled water (30 ml).

### Recovery of target metals from model solution and catalyst production

The seeded cells (1 wt%, 2 wt% or 5 wt% by mass of Pd, or Pt as specified; 16 mg of preloaded cells) were exposed to a mixed solution of 0.34 mM Pt(IV) and 0.42 mM Pd(II) in HNO_3_ (‘target metal’ solution; chosen as an approximation to a real catalyst leachate: Murray, 2012) to give a series of samples as shown in Table 1.

H_2_ was bubbled into the solution as before with reduction monitored in withdrawn samples as above with the results expressed as percentage of target metal reduced against time. Five independent batches were used for each test to check interbatch reproducibility. Following complete metal reduction, the cells were harvested by centrifugation, washed once in H_2_O and once in acetone, dried and ground in an agate mortar. The resulting powder was passed through a 100‐μ sieve to obtain a fine powder catalyst.

### Electron microscopy

Pellets of seeded bacteria (5 wt% Pd or Pt) were washed with distilled water, fixed (25% wt/vol glutaraldehyde) centrifuged, resuspended in 1.5 ml of 0.1 mM Na‐cacodylate buffer (pH 7) and stained with 1% osmium tetroxide in 0.1 M phosphate buffer pH 7; 60 min (Pd and mixed metal samples) or left unstained (Pt samples). Cells were dehydrated in an ethanol series (70, 90, 100, 100, 100% ethanol; 15 mins each) and washed twice in propylene oxide (15 min, 9500 g), embedded in epoxy resin, and the mixture was left to polymerize (24 h; 60°C). Sections (100‐150 nm) were cut from the resin block, placed on copper grids and viewed using a JEOL 1200CX2 TEM, accelerating voltage 80 kV.

### Catalytic evaluation via reduction of Cr(VI) to Cr(III)

Catalyst prepared as described above (Table 1; 10 mg of catalyst) was added to a 12‐ml serum bottle, and 5 mL of 0.5 mM Na_2_CrO_4_.4H_2_O in 20 mM MOPS‐NaOH buffer pH 7.0 was added. The bottle was sealed with a butyl rubber stopper, degassed under vacuum via a syringe, sparged with oxygen‐free nitrogen and placed onto a rotary shaker (180 rpm; 10 min) to ensure mixing and distribution of catalyst. Sodium formate (1 ml of a 25 mM solution) was added to initiate the reaction, and the shaken mixture (under N_2_) was sampled at 30‐min intervals. Centrifuged sample supernatants were analysed for residual Cr(VI) using diphenylcarbazide (Creamer *et al*., 2008).

### Catalytic evaluation in hydrogenation of soybean oil

Hydrogenation tests were as described by Zhu *et al*. (2016). Where different metal loadings/compositions were compared the mass of metal per test was maintained constant (no allowance was made for the different atomic weights of Pd and Pt). Analysis of products was as described by Zhu *et al*. (2016). Liquid samples were derivatized to fatty acid methyl esters (Christie and Han, 2010) for determination by gas chromatography (GC) [Varian CP‐3380 gas chromatograph with a flame ionization detector (FID) with a 75 m SP^TM^ 2560 capillary column (Sigma‐Aldrich, Irvine, UK)]. The temperature programme used initial temperature 200 °C, equilibration time of 5 min and temperature gradient of 4°C min^−1^ to 240 °C (30 min). The catalysts were assessed in terms of conversion of C18:2 (linoleic acid; ~50% of the starting material by assay), and selectivity was calculated as moles of oleic acid (*cis* C18:1) moles of all products detected^−1^ (Zhu *et al*., 2016).

## Conflict of interest

None declared.

## Supporting information


**Fig. S1.** High resolution electron microscopy study of palladium nanoparticle deposition in *E. coli* at 5 wt% Pd(0). High resolution STEM studies used a FEI image Cs‐corrector configuration TitanTM G2 60‐300 STEM microscope (300 kV: Omajali et al. 2015). Metallized cells (fixed, stained and sectioned) were viewed in STEM mode (B), via electron backscattering (A), and using HAADF‐STEM (High‐Angle Annular Dark Field‐Scanning Transmission Electron Microscopy: C) with EDX (Energy Dispersive X‐ray Spectroscopy) for Pd‐mapping (D).
