# Supplementary material for: Biorefining of platinum group metals from model waste solutions into catalytically active bimetallic nanoparticles
Source: Microb Biotechnol. 2017 Dec 28;11(2):359–68. doi: 10.1111/1751-7915.13030 (PMC5812250; doi:10.1111/1751-7915.13030)
Supplement: Supplementary file 1 — Fig. S1. High resolution electron microscopy study of palladium nanoparticle deposition in E. coli at 5 wt% Pd(0). High resolution STEM studies used a FEI image Cs‐corrector configuration TitanTM G2 60‐300 STEM microscope (300 kV: Omajali et al. 2015). Metallized cells (fixed, stained and sectioned) were viewed in STEM mode (B), via electron backscattering (A), and using HAADF‐STEM (High‐Angle Annular Dark Field‐Scanning Transmission Electron Microscopy: C) with EDX (Energy Dispersive X‐ray Spectroscopy) for Pd‐mapping (D). [file MBT2-11-359-s001.doc]

Supplementary information

High resolution electron microscopy study of palladium nanoparticle deposition in *E. coli* at 5 wt% Pd(0)


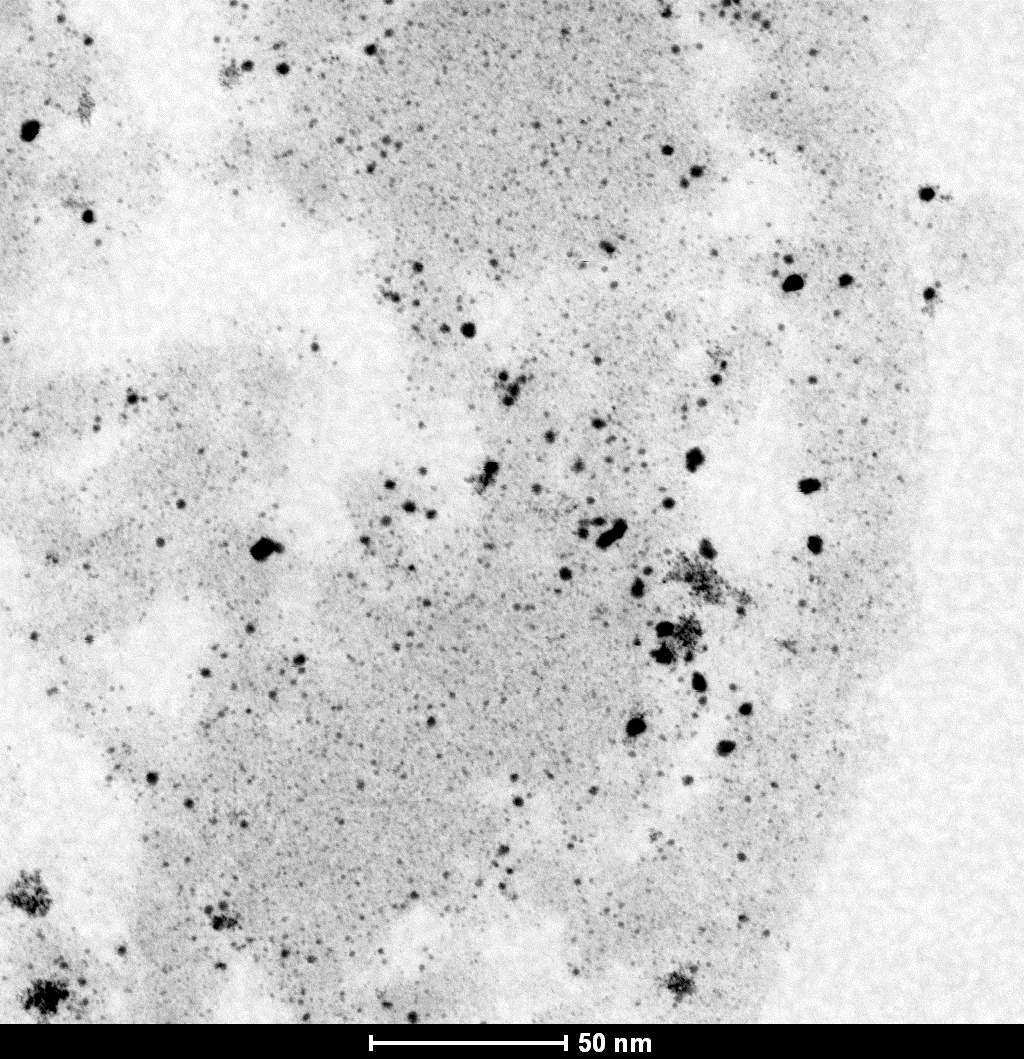


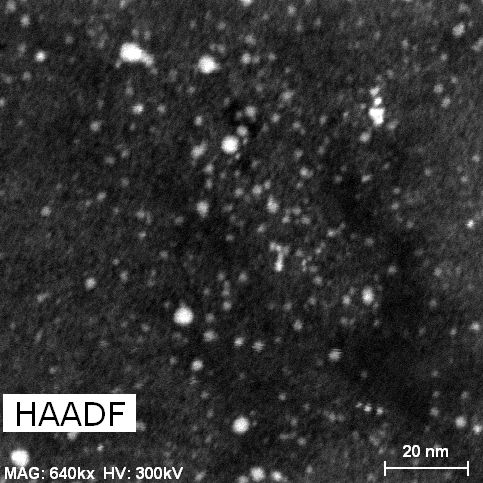


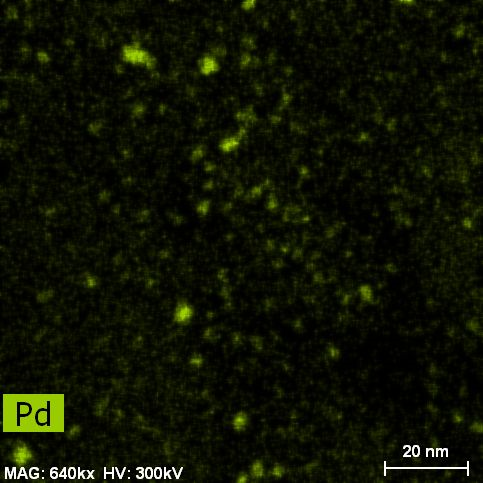


High resolution STEM studies used a FEI image Cs-corrector configuration TitanTM G2 60-300 STEM microscope (300 kV: Omajali et al. 2015). Metallized cells (fixed, stained and sectioned) were viewed in STEM mode (B), via electron backscattering (A), and using HAADF-STEM (High-Angle Annular Dark Field-Scanning Transmission Electron Microscopy: C) with EDX (Energy Dispersive X-ray Spectroscopy) for Pd-mapping (D)

**A**

**C**

**B**

**D**

(J. Bolivar-Gomez, I.Mikheenko


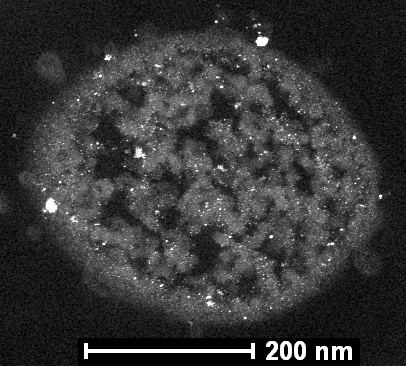

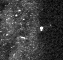


and L.E. Macaskie, unpublished)
